# Language as a barrier to colorectal cancer screening in Flanders: an ecological study

**DOI:** 10.1186/s13690-025-01541-3

**Published:** 2025-03-25

**Authors:** Simon Van den bergh, Lidia Casas, Gökhan Ertaylan, Guido Van Hal, Jos Bessems

**Affiliations:** 1 Environmental Intelligence Unit, Flemish Institute for Technological Research (VITO), Industriezone Vlasmeer 5, 2400 Mol (BE), Belgium; 2 Department of Family Medicine and Population Health (FAMPOP), Social Epidemiology and Health Policy (SEHPO), University of Antwerp, Universiteitsplein 1, 2610 Wilrijk (BE), Belgium; 3 Institute for Environment and Sustainable Development (IMDO), University of Antwerp, Universiteitsplein 1, 2610 Wilrijk (BE), Belgium; 4 Laboratory of Applied Microbiology and Biotechnology (LAMB), Department of Bioscience Engineering, University of Antwerp, Groenenborgerlaan 171, 2020 Antwerp (BE), Belgium; 5 Centre for Cancer Detection (CvKO), Research and Development, Ruddershove 4, 8000 Bruges (BE), Belgium

**Keywords:** Colorectal cancer, Cancer screening, Language, Ecological study, Flanders, Belgium

## Abstract

**Background:**

Despite its potential with regard to the prevention and early detection of colorectal cancer (CRC), participation in the organized CRC screening programme of the Belgian region of Flanders is suboptimal. The role of language discordance as a determinant of screening participation in Europe is poorly understood, despite being identified as a potential barrier in qualitative and non-European studies.

**Methods:**

In an ecological study analysing data on the level of Flemish municipalities (*n* = 300) from 2016 to 2021, we investigated whether the proportion of non-Dutch speakers at home is correlated with the response rate to CRC screening programme invitations and/or the total CRC screening coverage using multiple linear regression. We also performed Kruskal-Wallis tests and Dunn’s tests to examine municipal differences in screening based on their adjacency to the regions of Brussels and Wallonia.

**Results:**

After adjusting for confounders, the proportion of secondary school pupils that primarily speak a language other than Dutch at home was associated with a lower screening response rate (β = -0.327, 95% CI -0.359; -0.296)) and lower total screening coverage (β = -0.195, 95% CI -0.219; -0.171). Response rates and coverage were higher in municipalities at least two municipalities away from the border with Wallonia, Brussels or France. Our findings suggest that a high proportion of French speakers is particularly indicative of linguistic barriers to screening in Flemish municipalities (β = -0.358, 95% CI -0.397; -0.319 for response rate and β = -0.213, 95% CI -0.238; -0.188 for total coverage).

**Conclusion:**

Our study highlights the need to consider potential linguistic challenges when optimizing CRC screening policies.

**Supplementary Information:**

The online version contains supplementary material available at 10.1186/s13690-025-01541-3.

**Table:** 

Text box 1. Contributions to the literature
• Language barriers to colorectal cancer (CRC) screening have only been studied qualitatively or in settings outside of Europe.
• This study demonstrates that, in a Dutch-speaking region, the proportion of non-Dutch speakers is negatively correlated with CRC screening coverage and screening response rate on the municipal level.
• This study suggests that language is a barrier to CRC screening independent of citizenship at birth.
• This study observed lower CRC screening response and coverage in municipalities adjacent to areas with a different primary language but not in municipalities adjacent to a country with the same primary language as the study area.

## Introduction

Colorectal cancer (CRC) is the second most common type of cancer and the second leading cause of cancer death in Europe, according to recent estimates by the International Agency for Research on Cancer (IARC) [1, 2]. Early detection of CRC significantly improves a patient’s chances of survival, and most high-income countries have implemented some form of government-funded CRC screening programme to reduce CRC mortality [3–6]. Several studies also observed a reduction in CRC incidence in screened subpopulations, most noticeable in the incidence of advanced-staged cancers [2, 3, 6, 7]. A causal relationship between post-screening colonoscopy and decreased CRC incidence is plausible due to the ability of endoscopists to remove precancerous polyps during the procedure [8].

Cancer screening is a regional policy domain in Belgium, meaning there is no single nation-wide programme. The Flemish Region, colloquially known as Flanders, implemented its CRC screening programme in 2013. At first only people aged 66–74 years were invited, but the lower age threshold was gradually lowered over the years and has been 50 years of age since 2020 [3]. The upper threshold has remained unchanged. The Flemish Cancer Detection Centre (CvKO; *Du*: Centrum voor KankerOpsporing) sends one faecal immunochemical test (FIT) kit with instructions and a participation form to inhabitants of Flanders that have a Belgian social security number, are of eligible age, and do not fulfil any exclusion criteria. Submitting a stool sample via the organized programme is recommended every two years. Exceptions to this interval and exclusion criteria are described in more detail under “Outcomes, Exposures and Covariates” below. Participants typically receive their test result within 14 days, and those with positive (≥ 15 µg fHb/g) samples are referred for colonoscopy.

In Flanders as well as other European countries and administrative regions that have implemented an organized screening programme, participation in this programme is suboptimal [9]. Additionally, estimates of participation in FIT-based screening are heterogeneous across studies and study populations, with Mosquera et al. (2020) identifying a range of 2.3–68.7% participation in their systematic review [10]. Research by the CvKO has identified several demographic (e.g. foreign nationality), socio-economic (e.g. income) and behavioural (e.g. having had a GP visit in the past year) factors associated with participation in FIT-based CRC screening in Flanders [11, 12]. Qualitative evidence both from within Flanders [13, 14] and from other European settings [15–19] suggests that language may also be such a factor, but confirmation from quantitative studies is lacking [20]. However, limited English proficiency is associated with fewer colonoscopies and fewer faecal occult blood samples in the United States [21, 22].

The Belgian *Bestuurstaalwet* (*Eng*: Governing Language Law) of 1966 requires all official communication from the regional governments to be published or distributed exclusively in that region’s primary language [23], at least by default. Thus, screening invitations in Flanders are distributed only in Dutch. Twelve Flemish municipalities permit certain exceptions called “language facilities” to the aforementioned law, such as CRC screening test results being sent in French upon request [24].

Language discordance between the screening invitation and its recipient may occur in a diverse group consisting of French-speaking Belgians originally from Wallonia or the Brussels Capital Region, affluent and highly educated expats working for European institutions and other international organizations, recently arrived migrant workers, asylum seekers, and others. Unlike these groups, short-term visitors to Belgium and migrants in transit are not recorded in Belgium’s National Register, lack a Belgian social security number and are therefore not considered for government-funded CRC screening in Flanders [25]. We hypothesize that the response rate to Flemish CRC screening invitations is lower in municipalities that have a higher proportion of inhabitants that do not speak Dutch at home. We attempt to deepen our understanding of the effect language has on screening coverage and response rates by examining specific language groups as well as the effect of the proximity of Flemish municipalities to other language areas within Belgium.

## Materials & methods

### Study design & setting

We conducted a retrospective ecological study with the municipal level as the unit of interest. Our study population comprises all 300 (as of the 1st of January 2019) municipalities in the Flemish Region, the Dutch-speaking northernmost region of Belgium. The municipalities had a median population size of 15,125 (range: 78–529,417) inhabitants and a median population density of 414.50 (range: 51–3365) inhabitants per km² in 2021. Seven municipalities are the result of a municipal fusion ratified between 2017 and 2019. All data has been recalculated by the data owners to reflect these fusions, and only these recalculated data were analysed.

Our dataset comprises data from 2016 up to and including 2021 so that all variables of interest were represented in at least two separate years. The study was reported according to the STROBE guidelines [26] (Table S1).

### Data sources

Data on screening response and coverage rates were extracted from the online open-source database bevolkingsonderzoek.incijfers.be in late February 2024 [27]. Data on language spoken at home and data on covariates (age distribution, proportion of jobseekers, median income etc.) were extracted from the online open-source database provincies.incijfers.be in late February and early March 2024 [28]. The metadata are freely accessible on these respective platforms, though the platforms and the metadata are only available in Dutch.

Shapefiles of Belgian administrative units including the Flemish municipalities were downloaded from the online portal of the Belgian Federal Public Service Finance (coordinate reference system EPSG:3812 - ETRS89 / Belgian Lambert 2008) [29].

### Outcomes, exposures and covariates

We investigated two main outcomes related to CRC screening: CRC screening response rate and total CRC screening coverage, both expressed as proportions. For both variables, the denominator is the number of people of screening age living in the municipality on January 1 of a given year. The numerator for the response rate is the number of people of eligible age who are screened within 1 year of receiving an invitation for the Flemish programme. The numerator for the total coverage includes all the people included in response rate in addition to people screened outside of the organized programme and people who fulfil at least one exclusion criterion of this programme. Exclusion criteria for CRC screening are a full colectomy, a CRC diagnosis within the past 10 years, a full colonoscopy in the past 10 years, a virtual colonoscopy in the past 4 years, or a completed FIT test in the past 2 years.

The main exposure of interest was the proportion of secondary school pupils who do not speak Dutch at home. For households consisting of the pupil and three other members (with siblings counted together as one), pupils who speak Dutch with at most one other member are also counted in this variable’s numerator. We chose secondary school pupils rather than primary school pupils because we expect their parents to be older and more likely to be at or approaching the eligible screening age.

For the sub-analysis of specific language groups, the independent variable is the number of children 0–2 years of age living in a Flemish municipality that are spoken to in a language of interest by their mothers, divided by the total number of children in that age group living in the same municipality. The languages were grouped into the following categories: Dutch, French, Germanic (English & German), Eastern European (Polish, Romanian & Russian), Iberian Romance (Spanish & Portuguese), North African & Middle Eastern (Arabic, Turkish & Berber), and other (all others).

Municipalities were categorized into mutually exclusive groups according to their proximity to the Brussels and Walloon borders. We defined six categories. “Borders Wallonia” (*n* = 35) consists of all municipalities adjacent to the Walloon border. “Near Wallonia” (*n* = 38) comprises all municipalities adjacent to “Borders Wallonia” municipalities except the ones that border Brussels, which are categorized as “Borders Brussels + Near Wallonia” (*n* = 4). “Borders Brussels” (*n* = 8) comprises all remaining municipalities that border Brussels but not Wallonia. “Near Brussels” (*n* = 11) comprises all municipalities adjacent to at least one “Borders Brussels” or “Borders Brussels + Near Wallonia” municipality. All other municipalities (*n* = 204) were classified as “Far” (Table S2).

Factors identified as correlates of screening participation were collected as covariates. These include age, sex, citizenship at birth, socio-economic status (collected as occupational status, educational attainment, median income and child deprivation), comorbidities (collected as inhabitants with chronic diseases and inhabitants with disabilities), and health-seeking behaviour (collected as inhabitants with a global medical record and inhabitants with at least one doctor visit per year) [11, 12, 30, 31]. We additionally considered the year, population density, presence of local service centres (meant to stimulate self-sufficiency and social cohesion in the elderly), number of patients per GP practice and the existence of language facilities as covariates. See Table S2 in the supplementary material for more information on these variables.

### Statistical analysis

We calculated Spearman’s rank correlation coefficients to quantify correlations between exposures or covariates on the one hand and the outcome measures on the other hand. We fitted multiple linear regression models for both outcome variables by iteratively adding variables based on these Spearman coefficients and their significance levels until the model achieved optimal fit based on its Akaike information criterion (AIC). For these models, the variance inflation factor (VIF) for the variable “proportion of secondary school pupils who do not speak Dutch at home” was not allowed to be ≥ 5. Thus, these models are henceforth referred to as the “low-VIF models”. Subsequently, similar linear regression models were fitted for each specific language group. If data was missing for any modelled variable for a given municipality and a given year, that year-municipality pair (e.g. “Zuienkerke 2019”) was excluded from analysis. Although neither the exposure nor the outcome variables are normally distributed, the output of diagnostic plots for linear regression models (residuals vs. fitted plot, QQ-plot, scale-location plot, and residuals vs. leverage plot) did not discourage the use of this type of model.

The performances of municipalities with different national border statuses with regard to the two screening indicators were compared using Kruskal-Wallis and post-hoc Dunn’s tests.

All statistical analyses were performed in R v4.3.2. The initial significance level was set at α = 0.05, but a Bonferroni correction was applied to the correlation matrix (α = 1.15E-04).

### Secondary & sensitivity analyses

Since the construction of the low-VIF models was solely based on the correlation coefficients of the considered covariates with the exposure of interest and on the AIC of the resulting model, it does not take into account whether the included covariates were confounders or effect modifiers. A directed acyclic graph (DAG) containing all exposures, outcomes and collected covariates was constructed using the online tool DAGitty to address this (Figure S1). This DAG’s pathways are based on the aforementioned Spearman coefficients as well as the literature and the researchers’ judgment. We constructed multiple linear regression models for each outcome variable based on the sole combination of variables that, when conditioned for, left no confounding paths open in the DAG. These models are henceforth referred to as the “DAG-compliant models”.

We analysed both the low-VIF models and DAG-compliant models while excluding the decile of municipalities with the lowest population size, since small fluctuations in such towns could otherwise impact our results considerably. We also performed the analyses on a subset of data from 2016 to 2019 to account for possible effects of the COVID-19 pandemic. In another set of sensitivity analyses we excluded all municipalities bordering another country (France or the Netherlands) or region (Brussels or Wallonia), and all municipalities bordering Francophone countries or regions (France, Brussels or Wallonia), respectively. Finally, we analysed the DAG-based model for a subset of data from 2017, the last year for which data on educational attainment was available, and the low-VIF model for a subset of data from 2021, the most recent year in our dataset.

National and international border statuses were coded in “Borders”, “Near” and “Far” categories for the proximity to Wallonia, Brussels, the Netherlands and France separately. Each municipality was also given a “Francophone border score” for which it received 2 points for adjacency to each of the aforementioned areas minus the Netherlands, 1 point for being one municipality removed from them, and no points for being at least two municipalities removed from them.The method for border status analysis described earlier was repeated to investigate potential differences between municipalities in the “Borders”, “Near” and “Far” groups for each of the areas adjacent to Flanders (Brussels, Wallonia, the Netherlands, France). Finally, the relationship between the Francophone border score and the screening metrics was assessed by calculating Spearman’s rank correlation coefficients and their associated 95% confidence intervals (CIs), the latter of which were calculated using the method described by de Carvalho & Marques (2012) [32].

## Results

### Description of data

**Fig. 1:**
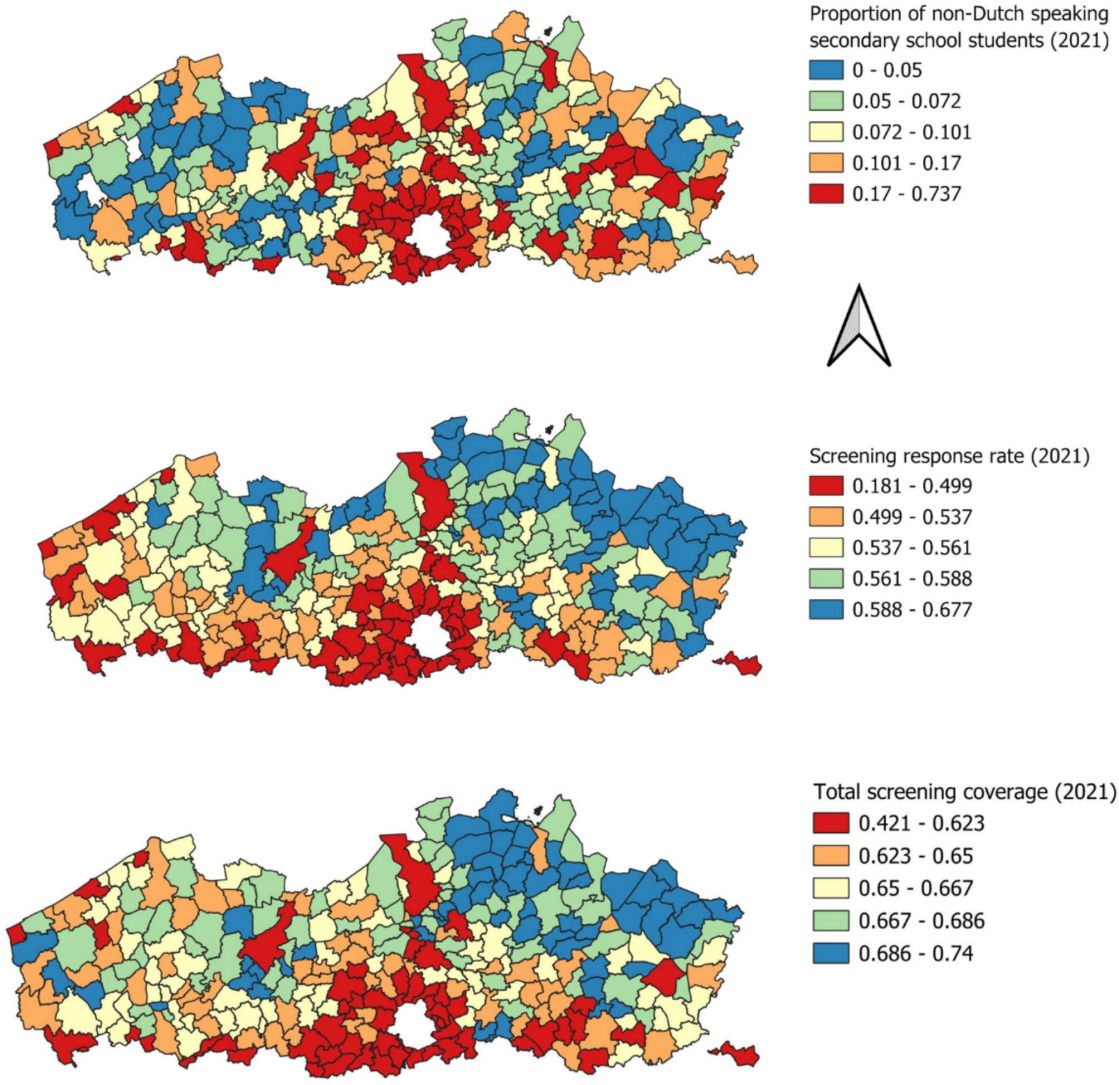
Geographical distribution of non-Dutch speaking secondary school students (top), screening response rate (centre), and total screening coverage (bottom) quintiles on the municipal level in Flanders in 2021

Figure 1 shows the geographical distribution of the proportion of secondary school pupils who do not speak Dutch at home, screening response rate, and total screening coverage quintiles in Flanders for 2021, while Table S3 summarizes the descriptive statistics of all non-categorical variables considered for analysis.

The proportion of secondary school pupils that do not speak Dutch at home ranged from 1.0 to 73.7% in Flemish municipalities, with a mean of 10.8% and a median of 7.0%. This percentage has been steadily increasing across the whole of Flanders in recent years (Figure S2 top). Information on this variable was missing or censored in 21 Year-Municipality combinations and was entirely non-quantifiable in one municipality; these missing records were excluded from all analyses where this variable was the main exposure. While Dutch is still the most common language spoken to young children in Flanders, its prevalence has decreased in 2023 compared to 2013 (Table S4; data from Kind & Gezin). The proportion of children addressed in French, Arabic and Romanian increased during that time period, while the proportion of children addressed in Turkish and Berber decreased. Aggregated data on children 0–2 years of age addressed in a specific language by their mothers were only available from 2020 onward, but no municipalities had missing data for any of the categories. Table S3 and Figure S3 reveal that the proportion of secondary school pupils not speaking Dutch at home is positively skewed. This is also observed for the language subgroups, except Dutch (which is negatively skewed), with positive skewness being highest in the proportion of French speakers. Shapiro-Wilks tests on the distributions of the exposure variables showed that none were normally distributed (*p* < 0.0001 for each variable).

Total screening coverage ranged from 38.4 to 78.2%, with a mean of 65.9% and a median of 66.6%. Screening coverage is notably lower in the more recent years of the study period compared to 2016–2017 (Figure S2 centre) but does not seem to have decreased further during the COVID-19 years. No records had missing or censored data for this variable. Response rates ranged from 13.6 to 70.3%, with a mean of 52.9% and a median of 53.9%. The mean Flemish response rate remained relatively constant over the last five years with the notable exception of 2020, the first year of the COVID-19 pandemic, where the overall response rate decreased to slightly below 50% (Figure S2 bottom). No records had missing or censored data for this variable. Neither outcome variable is normally distributed (*p* < 0.0001) and both distributions show negative skew.

Table S5 summarizes the distribution and descriptive statistics of the 300 Flemish municipalities according to their border status. Most municipalities (204 out of 300) are located at least two municipalities away from any Francophone border, with 35 municipalities bordering Wallonia, 15 bordering Brussels, and 10 bordering France. A total of 35 municipalities border the Netherlands. The only other categorical variable included in this study is the presence of language facilities, which 12 out of 300 Flemish municipalities have. None of the categorical variables had missing data.

### Crude correlation coefficients

A matrix of Spearman’s rank correlation coefficients for each two-by-two correlation test of variables can be found in the supplementary material (Table S6). After Bonferroni correction, screening response rate was positively correlated with total screening coverage, all age variables, proportion of males, proportion of inhabitants with secondary education as highest level, proportion of inhabitants with a global medical record, proportion of inhabitants who visited a doctor at least once in the past year, number of patients per GP practice and proportion of Belgians at birth while it was negatively correlated with year, proportion of secondary school pupils who do not speak Dutch at home, proportion of financial aid recipients, population density, median net taxable income, child deprivation, and the proportion of inhabitants who were non-EU citizens at birth. Total screening coverage showed the same correlation pattern with the aforementioned variables, in addition to being positively correlated with the proportion of people in retirement and negatively with the proportion of inhabitants who were non-Belgian EU citizens at birth.

**Fig. 2:**
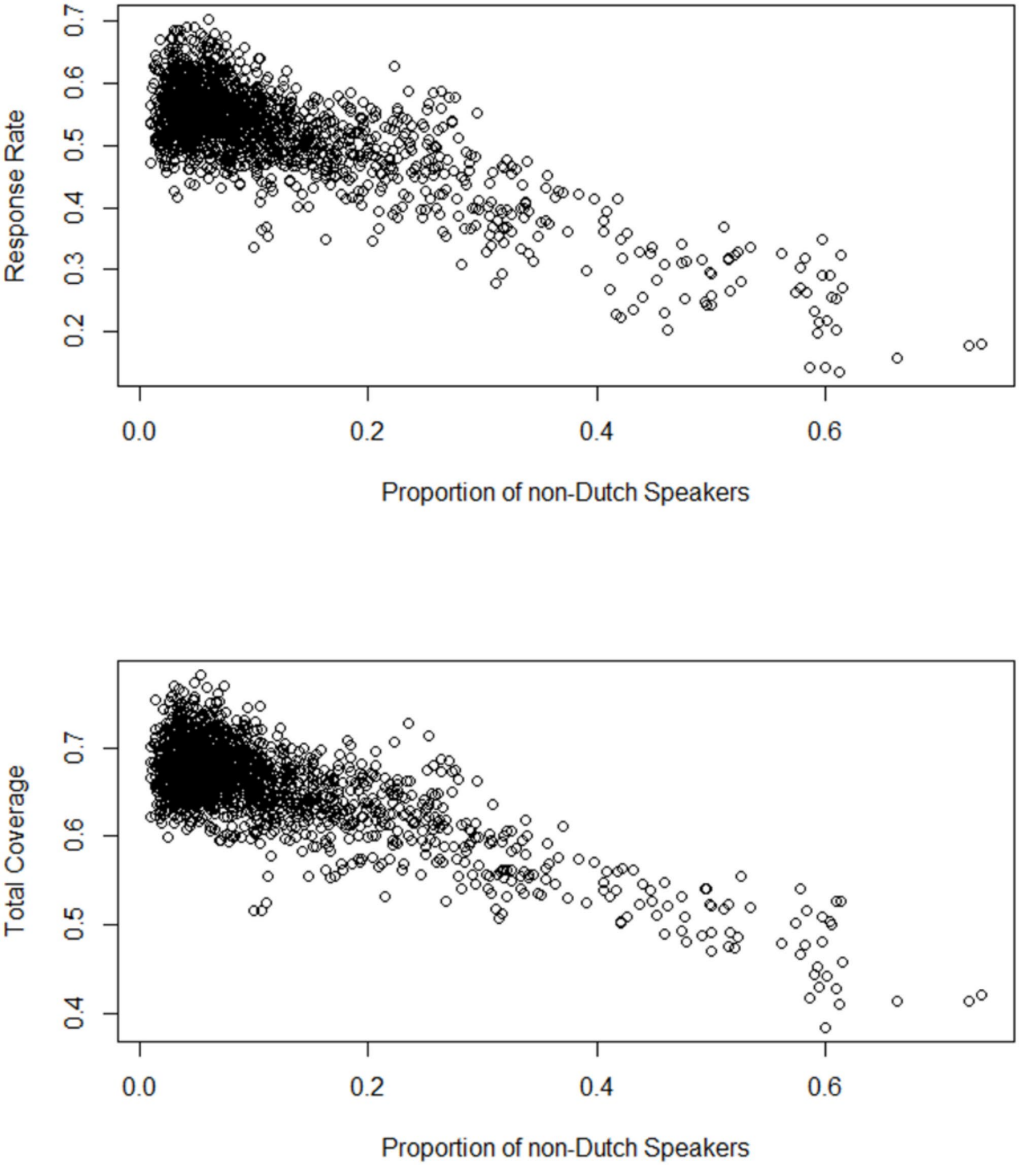
Scatterplots of screening response rate (top) and total screening coverage (bottom) by the proportion of secondary school pupils who do not speak Dutch at home; all years (2016–2021) combined. (*n* = 1779)

Figure 2 shows a scatterplot of the proportion of secondary school pupils who do not speak Dutch at home plotted against the outcome variables screening response rate and total screening coverage.

### Language & screening regression models

The low-VIF model for screening response rate and the proportion of pupils that do not speak Dutch at home (*n* = 1775) contained the following covariates: national border status, proportion of inhabitants with at least one doctor visit in the past year, proportion of 55-59-year olds, proportion of 60-64-year olds, proportion of 65-69-year olds, proportion of males, language facilities, proportion of inhabitants with at least one chronic disease, proportion of inhabitants that are only wage-earners, proportion of financial aid recipients, proportion of inhabitants in (early) retirement, and median net taxable income. The proportion of secondary school pupils who do not speak Dutch at home was inversely related to screening response rate (β = -0.327, 95% CI -0.359; -0.296) independent of these covariates.

The low-VIF model for total screening coverage and the proportion of pupils that do not speak Dutch at home (*n* = 1775) contained the following covariates: national border status, proportion of 50-54-year olds, proportion of 55-59-year olds, proportion of 65-69-year olds, proportion of 70-74-year olds, language facilities, proportion of inhabitants with a global medical record, year, population density, patients per GP practice, proportion of inhabitants in (early) retirement, and median net taxable income. The proportion of secondary school pupils who do not speak Dutch at home was inversely related to total screening coverage (β = -0.195, 95% CI -0.219; -0.171) independent of these covariates.

When we instead studied the proportion of children 0–2 years of age addressed in a specific language or language group (data from 2020 to 2021 only) in a multiple linear regression model adjusting for the same covariates, we observed a positive association between the proportion of children addressed in Dutch at home and the response rate to screening invitation (β = 0.137, 95% CI 0.103; 0.171), and a positive association between children addressed in Polish, Romanian or Russian and response rate. A negative association with this outcome variable was found for the proportion of children addressed in French (β = -0.301, 95% CI -0.257; -0.345) or Germanic languages. Non-significant results were obtained for Iberian Romance, North African & Middle Eastern, and other languages (Table 1).

We found a positive association between the proportion of children addressed in Dutch and total screening coverage (β = 0.104, 95% CI 0.081; 0.127) as well as for Eastern European languages. In contrast, the proportion of children addressed in French (β = -0.201, 95% CI -0.232; -0.170), Germanic languages, North African & Middle Eastern languages or uncategorized “other” languages was negatively associated with total coverage. The association with Iberian Romance languages was not significant for coverage. All regression coefficients with their 95% CIs are summarized in Table 1, while Figure S4 shows scatterplots of the proportions for each language group against response rate and total coverage.

**Table 1:** Beta coefficients and corresponding 95% CIs of multiple linear regression models with the proportion of children 0–2 years of age addressed in a specific language by their mothers as the independent variable of interest. (*n* = 598)

Language Group	β (95% CI)	
	*Response Rate*	*Total Coverage*
*Dutch*	0.137 (0.103; 0.171)	0.104 (0.081; 0.127)
*French*	-0.301 (-0.345; -0.257)	-0.201 (-0.232; -0.170)
*German*,* English*	-0.466 (-0.754; -0.178)	-0.364 (-0.570; -0.158)
*Russian*,* Polish*,* Romanian*	0.115 (0.003; 0.127)	0.114 (0.033; 0.195)
*Spanish*,* Portuguese*	-0.063 (-0.433; 0.307)	0.123 (-0.147; 0.393)
*Arabic*,* Turkish*,* Berber*	-0.024 (-0.101; 0.053)	-0.113 (-0.162; -0.063)
*Other*	0.091 (-0.012; 0.194)	-0.107 (-0.166; -0.048)

All models were adjusted for border status, language facilities (except response rate– French), proportion of 55-59-year olds, proportion of 65-69-year olds, proportion of retired inhabitants, and median income. Response rate models were additionally adjusted for the proportion of inhabitants with a doctor visit in the past year and proportion of wage-earners, and for deprivation index in all except Dutch. The model for Dutch was also adjusted for the proportion of financial aid recipients and the proportion of 60-64-year olds, and the models for Dutch and “other” languages were adjusted for the proportion of inhabitants with chronic diseases. Coverage models were additionally adjusted for the proportion of 70-74-year olds, for global medical record, year and patients per GP practice in all except French, and for population density in all except Dutch. Finally, the response rate models for Dutch, French, German/English and North African & Middle Eastern languages, as well as the coverage model for French, were adjusted for sex

### Comparisons between municipalities by border status

Kruskal-Wallis tests revealed significant differences in screening response rate and total screening coverage between national border status categories (*p* < 2.2E-16 for both outcomes), though differences in proportions between groups tend to be smaller for total coverage than for response rate (Table 2). Results of the subsequent pairwise Dunn’s tests are summarized in Tables 3 and 4.

**Table 2:** Descriptive statistics for border status categories of national borders. (*n* = 1800)

National Border Status	Municipalities (*N*)	Min.	Q1	Median	Mean	Q3	Max
*Screening Response Rate*							
Far From Border	205 (1230)	0.383	0.526	0.556	0.556	0.587	0.703
Near Brussels	11 (66)	0.256	0.453	0.484	0.473	0.519	0.575
Borders Brussels	8 (48)	0.198	0.297	0.333	0.332	0.386	0.448
Borders Brussels + Near Wallonia	4 (24)	0.136	0.198	0.284	0.282	0.369	0.438
Near Wallonia	37 (222)	0.437	0.501	0.529	0.530	0.556	0.656
Borders Wallonia	35 (210)	0.230	0.421	0.475	0.462	0.509	0.636
*Total Screening Coverage*							
Far From Border	205 (1230)	0.559	0.656	0.675	0.676	0.698	0.782
Near Brussels	11 (66)	0.499	0.614	0.631	0.627	0.653	0.701
Borders Brussels	8 (48)	0.428	0.481	0.515	0.518	0.558	0.612
Borders Brussels + Near Wallonia	4 (24)	0.384	0.482	0.530	0.511	0.558	0.598
Near Wallonia	37 (222)	0.595	0.640	0.658	0.660	0.682	0.746
Borders Wallonia	35 (210)	0.507	0.574	0.618	0.614	0.653	0.721

**Table 3:** Results of pairwise Dunn’s tests comparing screening response rate between national border status categories. (*n* = 1800)

Z (*p*) Screening Response Rate	Far From Border	Borders Brussels	Borders Brussels + Near Wallonia	Borders Wallonia	Near Brussels
*Borders Brussels*	-13.178 (*< 0.0001*)				
*Borders Brussels + Near Wallonia*	-9.565 (*< 0.0001*)	0.130 (*1.000*)			
*Borders Wallonia*	-16.780 (*< 0.0001*)	-4.286 (*0.0001*)	-3.333 (*0.0064*)		
*Near Brussels*	9.808 (*< 0.0001*)	-3.688 (*0.0017*)	-3.071 (*0.0160*)	-0.099 (*1.000*)	
*Near Wallonia*	7.235 (*< 0.0001*)	-8.924 (*< 0.0001*)	-6.755 (*< 0.0001*)	-7.648 (*< 0.0001*)	-5.134 (*< 0.0001*)

**Table 4:** Results of pairwise Dunn’s tests comparing total screening coverage between national border status categories. (*n* = 1800)

Z (*p*) Total Screening Coverage	Far From Border	Borders Brussels	Borders Brussels + Near Wallonia	Borders Wallonia	Near Brussels
*Borders Brussels*	-13.084 (*< 0.0001*)				
*Borders Brussels + Near Wallonia*	-9.352 (*< 0.0001*)	0.0099 (*1.000*)			
*Borders Wallonia*	-15.934 (*< 0.0001*)	-4.595 (*< 0.0001*)	-3.423 (*0.0046*)		
*Near Brussels*	8.620 (*< 0.0001*)	-4.407 (*0.0001*)	-3.518 (*0.0033*)	-0.715 (*1.000*)	
*Near Wallonia*	6.207 (*< 0.0001*)	-9.304 (*< 0.0001*)	-6.896 (*< 0.0001*)	-7.762 (*< 0.0001*)	-4.589 (*< 0.0001*)

**Fig. 3:**
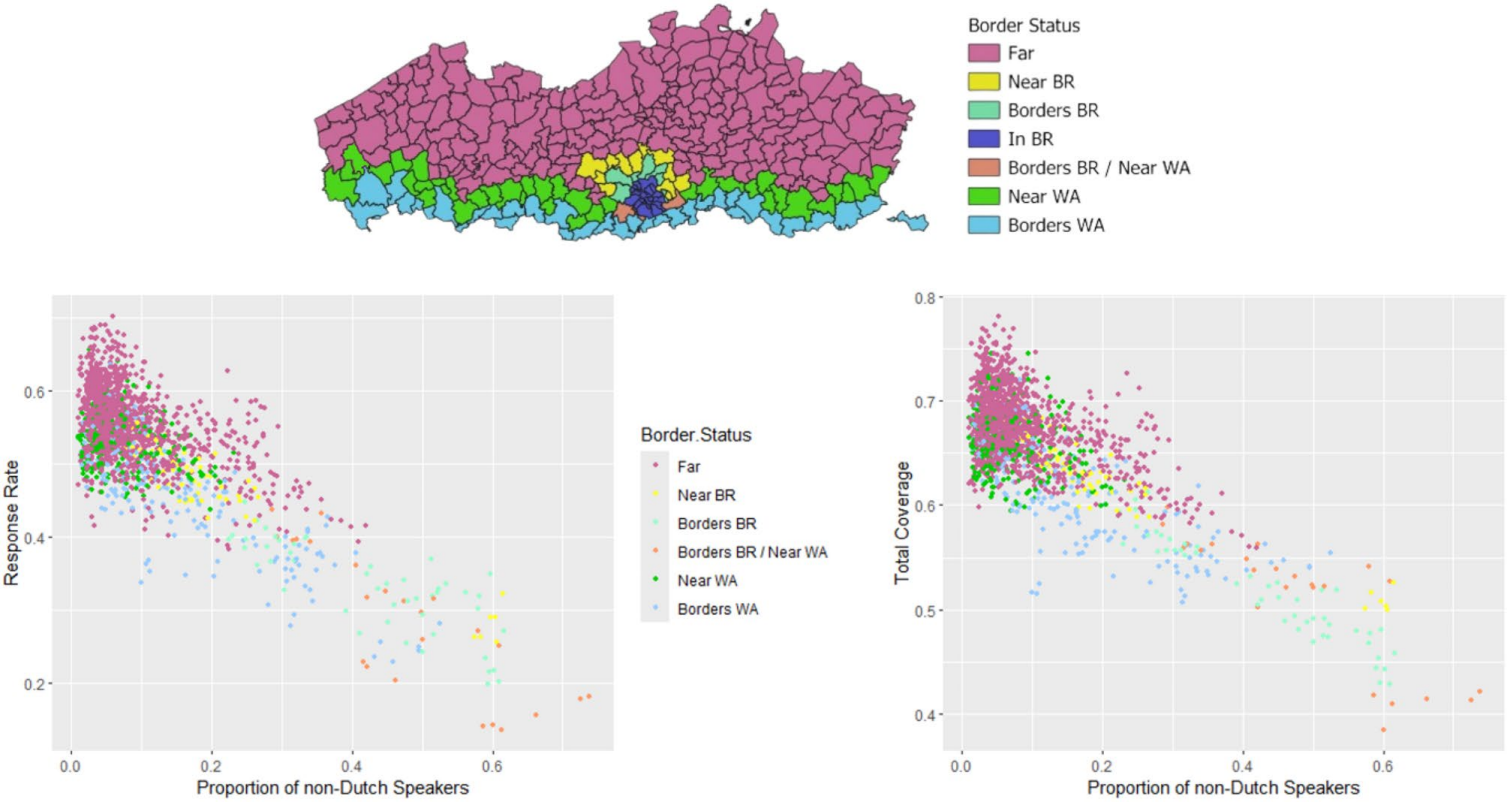
(Top) Distribution of Flemish municipalities according to their adjacency to Brussels and Wallonia. (Bottom) Scatterplots of the proportion of secondary school students who do not speak Dutch at home plotted against screening response rate (left) and total screening coverage (right) with data points coloured according to their national border status. (*n* = 1800)

The results are suggestive of screening coverage and response gradients across national border status categories, with municipalities adjacent to another Belgian region undergoing relatively less screening than municipalities “near” (but not adjacent to) that region which in turn have lower response rates and screening coverage than municipalities “far” from the border to that region (Fig. 3). The four municipalities bordering Brussels and one municipality away from Wallonia had the lowest screening response rates (median = 0.284, mean = 0.282) and mean total screening coverage (0.511). The median total screening coverage in municipalities bordering Brussels but not near Wallonia was lower still (0.515 compared to 0.530 for the Border Brussels + Near Wallonia group). However, the differences between these two categories was not significant for either screening indicator. By contrast, the screening response rates and coverages in municipalities at least two municipalities away from Brussels and Wallonia was significantly higher than in all other categories.

### Secondary & sensitivity analyses

For screening response rate, our final DAG-compliant model (*n* = 590) adjusted for the covariates proportion of non-Belgian EU-at-birth inhabitants, proportion of non-EU-at-birth inhabitants, proportion of 50-54-year olds, proportion of 60-64-year olds, proportion of 70-74-year olds, proportion of males, language facilities, year, population density, and the three proportions of educational attainment (primary, secondary and tertiary as highest, respectively). The inverse association between the proportion of pupils who do not speak Dutch at home and screening response rate was stronger in this model (β = -0.743, 95% CI -0.835; -0.651) compared to the low-VIF model.

For total screening coverage, our final DAG-compliant model (*n* = 590) adjusted for the covariates proportion of Belgian-at-birth inhabitants, proportion of 55-59-year olds, proportion of 60-64-year olds, proportion of males, language facilities, year, population density, and the three proportions of educational attainment. The inverse association between the proportion of secondary school pupils not speaking Dutch at home and total screening coverage was stronger in this model (β = -0.416, 95% CI -0.462; -0.370) compared to the low-VIF model.

The negative associations between pupils not speaking Dutch at home and the outcome variables were slightly stronger when only the 90% most populous municipalities were considered for both response rate (β = -0.359, 95% CI -0.391; -0.327 for the low-VIF model and β = -0.792, 95% CI -0.885; -0.699 for the DAG-compliant model) and total coverage (β = -0.214, 95% CI -0.239; -0.189 for the low-VIF model and β = -0.440, 95% CI -0.486; -0.394 for the DAG-compliant model) compared to the analysis including all municipalities.

When only pre-COVID years were considered, the negative association between pupils not speaking Dutch and response rate was slightly stronger (β = -0.332, 95% CI -0.372; -0.293 for the low-VIF model) than in the analysis encompassing all years, as was the association between pupils not speaking Dutch and total coverage (β = -0.200, 95% CI -0.232; -0.168 for the low-VIF model). Since the DAG-compliant model is adjusted for education level (for which only data up to 2017 was available), it is restricted to pre-COVID years by default.

Excluding municipalities bordering another country or language region attenuated the association with response rate (β = -0.245, 95% CI -0.287; -0.203 for the low-VIF model and β = -0.497, 95% CI -0.660; -0.334 for the DAG-compliant model) and, to a lesser extent, with total coverage (β = -0.180, 95% CI -0.211; -0.149 for the low-VIF model and β = -0.373, 95% CI -0.456; -0.290 for the DAG-compliant model). This attenuation is less pronounced when only municipalities with Francophone borders were excluded, as seen in the regression coefficients of the models for response rate (β = -0.293, 95% CI -0.332; -0.254 for the low-VIF model and β = -0.526, 95% CI -0.684; -0.368 for the DAG-compliant model) and total coverage (β = -0.177, 95% CI -0.206; -0.148 for the low-VIF model and β = -0.324, 95% CI -0.385; -0.263 for the DAG-compliant model).

The inverse associations between the proportion of pupils who do not speak Dutch at home and screening response rate on the one hand (β = -0.337, 95% CI -0.411; -0.263) and total screening coverage on the other hand (β = -0.204, 95% CI -0.258; -0.150) in the low-VIF model were slightly stronger when only data from 2021 were considered. The associations also remained robust when analysing the DAG-compliant model with data from 2017 only, with a slightly less negative regression coefficient for response rate (β = -0.733, 95% CI -0.864; -0.602) and a slightly more negative coefficient for total coverage (β = -0.431, 95% CI -0.496; -0.366).

A secondary analysis of specific language groups using DAG-compliant models yielded correlations comparable to those of the low-VIF models (Table S7). The positive association between the proportion of children 0–2 years of age addressed in Dutch and screening response rate was stronger than in the corresponding low-VIF analysis (β = 0.342, 95% CI 0.305; 0.379). Likewise, the association between the proportion of children 0–2 years of age addressed in French and screening response rate was more strongly negative (β = -0.358, 95% CI = -0.397; -0.319). The same trend manifested for the total screening coverage, with a higher β coefficient for Dutch (β = 0.202, 95% CI 0.181; 0.223) and a lower coefficient for French (β = -0.213, 95% CI -0.238; -0.188).

When considering international borders in addition to regional borders, proximity to the Walloon, Brussels and French border follows a mostly unitary pattern of “Far” municipalities outperforming “Near” municipalities, which in turn outperform bordering municipalities (Tables S4 & S8). This finding is corroborated by the correlation analysis between municipalities’ Francophone border scores and their screening performance; municipalities with a lower border score have higher response rates (ρ = -0.540, 95% CI -0.575; -0.505) and coverage (ρ = -0.482, 95% CI -0.521; -0.443) (Figure S5). By contrast, municipalities bordering or near the Dutch border have a higher screening response rate and screening coverage than municipalities farther away from the Netherlands.

## Discussion

### Framing within state of the art

We hypothesized that municipalities with a higher proportion of non-Dutch speakers have lower response rates to FIT-based screening invitations. Our study on Flemish municipalities found an inverse relationship between the proportion of inhabitants that do not speak Dutch at home and the screening response rate, as well as the total screening coverage. These findings support our main hypothesis and are in agreement with earlier research from Hoeck et al. (2019), who concluded that non-Belgian (or Dutch) citizenship at birth is associated with lower CRC screening uptake [33]. While language and citizenship are distinct variables, the proportions of Belgian, non-Belgian EU and non-EU citizens at birth were strongly correlated with the proportion of non-Dutch-at-home speakers in our study (Table S6). Our observations also corroborate qualitative studies that listed a lack of understanding due to language discrepancies as an impediment to screening adherence, not just in Belgium [13, 14] but across several European countries [16–19].

Total screening coverage includes opportunistic screening recommended by a medical expert such as a GP. Under the assumption of language concordance between most patients in Flanders and their GP, language would reasonably have a stronger effect on response rate than on total coverage. The regression coefficients of the total screening coverage models in the present study are almost always closer to the null than their corresponding response rate models, including in sensitivity analyses (exceptions being low-VIF response rate models where the association was not significant; see Table 1), affirming this expectation. This observation aligns with a study by Tran et al. (2022), who found that differences in screening rates within the government-funded programme are greater than differences in opportunistic screening in a selection of municipalities with a high proportion of native French speakers compared to rates in all Flemish municipalities combined [24].

If the main driver of screening disparities in municipalities with many non-Dutch-speakers is a preference for opportunistic screening or participation in another region, then these differences would be apparent in response rate but not in total coverage. If on the other hand, the dominant driver is low language proficiency or low health literacy, then differences in these drivers should be reflected in both response rate and coverage. The results of this study do not decisively point to either conclusion. Similar to the coefficients of the regression models, we found smaller but still significant differences between border status categories for coverage than for response rate. It is likely that the variability in screening outcomes across Flemish municipalities is not attributable to any one of the factors listed above, but rather a combination thereof.

Limited proficiency in English has been associated with fewer screening colonoscopies and fewer faecal occult blood samples in the United States [21, 34]. Cataneo et al. (2022) furthermore found lower screening rates in Spanish-speaking groups compared to people speaking other non-English languages [21]. Our study also found differences between specific languages or language groups, with the proportion of French speakers in particular being negatively associated with CRC screening. One study from California found a negative association between CRC screening adherence and speaking a language other than English at home among South Asian Americans, but not with language proficiency [35]. This suggests that at least in some populations, language may not represent a barrier to screening per se but rather a surrogate variable for unmeasured cultural factors.

Such cultural factors may, alongside a small overall number of speakers of certain languages (e.g., Iberian Romance languages), go some way toward explaining the inconsistent language-screening correlations found in our study. Research on CRC screening attitudes of ethnic minorities in European countries frequently cites embarrassment, lack of knowledge of CRC, and lack of trust in the health industry or health care system as important barriers [13, 18, 36, 37].

Nevertheless, negative associations between the proportion of children addressed in French and CRC screening were robust across models and across outcome measures in our study. Response rates and screening coverage were also lower in municipalities closer to French-speaking areas (Wallonia, Brussels or France) than in municipalities farther away from them, in contrast to municipalities bordering the Netherlands where these rates were comparatively higher. These differences in screening rates may be influenced by Francophone inhabitants who undergo screening in a French-speaking region they live close to.

Aside from Dutch, the proportion of Polish, Romanian and Russian speakers was also positively associated with CRC screening response and coverage on the municipal level in our study. Polish and Romanian citizens who migrate to Belgium primarily do or did so for a remunerated economic activity as reported by the Federal Centre for Migration (MYRIA) [38]. While the main motivators of Eastern European labour migration are better pay and working conditions [39], the availability of health care services may be seen as a secondary benefit. The evidence on this is conflicting, as one systematic review concluded that migrants are more likely to use emergency services or hospitalizations but less likely to use screening services [40]. It is possible that migrants from the EU in particular tend to compare the health care services offered in their home country and in their country of employment [41], and in the case of CRC screening decide that the Flemish programme is more favourable. It is worth noting that the organized Romanian screening programme was still in its pilot phase during our study period [42] while the primary screening modality in Poland is colonoscopy [43], which is often perceived as invasive and embarassing.

The non-significant association between the proportion of Arabic, Turkish and Berber speakers and the screening response rate is surprising, since previous research has shown that people from Türkiye or the Maghreb are less likely to undergo CRC screening in Flanders [33]. Native speakers of these languages are well-represented in former mining towns in the province of Limburg (probably due to a sizable Turkish diaspora consisting of former miners, their reunited families and their descendants) that generally perform better in terms of screening compared to the largest Flemish cities or the suburbs around Brussels where such speakers are also prevalent. Such diaspora may have very different priorities, levels of integration, levels of health literacy and attitudes towards cancer screening and follow-up procedures than other speakers of Middle Eastern and North African languages (e.g. recently immigrated Syrian refugees), but our ecological study is not well-suited to distinguish these different subpopulations.

In our analyses, language was more strongly correlated with the two screening variables than the socio-economic variables income, educational attainment, and child deprivation (Table S6, Fig. 2 & S6). We hypothesize that language affects cancer screening via an unambiguous mechanism, i.e. poor understanding of the information and recommendations provided by screening and primary care providers leads to lower participation. On the other hand, factors such as higher confidence in colonoscopy compared to FIT [44, 45], costs associated with and willingness to pay for opportunistic screening [11, 46], prioritization of one’s long-term health [14, 47], self-perceived risk based on one’s health-seeking behaviour and comorbidities [47–49], trust in health care institutions [13, 18, 50], peer encouragement [20], and health literacy [20, 51] may all contribute to a more complex mix of drivers of and impediments to organized screening across different socio-economic strata.

To our knowledge, this is the first paper to focus on language spoken at home with relation to CRC screening in a European population. We propose three potential explanations for this relatively meagre evidence base. First, research settings may lack high-quality data on language spoken at home. In the Netherlands, for example, these data have most recently been collected cross-sectionally in the context of a dedicated study in 2019, rather than routinely and annually like in Flanders [52]. Second, certain data collection tools exclude or discourage participation by people with low proficiency in the study setting’s native language, as is the case with written questionnaires that are only distributed in a single language [30]. Third, language as a variable may be excluded from analysis due to multicollinearity [53]. Multicollinearity was also observed in our study, with language and citizenship at birth showing a particularly strong correlation, but we opted to exclude the latter in our main (low-VIF) model instead.

### Strengths & limitations

A notable strength of this study is that it incorporated several demographic, behavioural and health-based factors that were previously identified as being correlated with CRC screening outcomes in Flanders while also adding new ones [11, 12]. The focus of this study is different from those previous publications, however, in that it examines a single exposure in depth rather than giving an overview of risk and protective factors.

Our study has several limitations. First, the ecological nature of our analysis means that ecological bias cannot be ruled out: we cannot draw conclusions on correlations between language spoken at home and screening outcomes on an individual level, nor can we infer causality. Even so, we have laid a foundation for studying the effects of language in specific areas of interest (e.g., communities bordering Wallonia and/or Brussels), which may be more efficient than a region-wide approach. Moreover, our results align with qualitative findings across Europe. Second, the variable “proportion of pupils who do not speak Dutch at home” is partially a misnomer since these percentages do include pupils in households of size three or more who speak Dutch with at most one member of this household. Pupils who speak Dutch with one parent but not the other nor with any of their siblings are thus counted as “not speaking Dutch”. While this variable serves as an indicator for the proportion of inhabitants that mainly speak a language other than Dutch, it does not measure the proficiency level of either the pupils or their parents. It also does not account for single-person households, which may have distinct screening profiles compared to members of larger households [12]. Third, most of the models that did not contain any confounding pathways according to our DAG had high (> 5) VIFs for the exposure of interest. This limitation was addressed by analysing two types of models: a low-VIF model that prioritized the circumvention of multicollinearity, and a DAG-compliant model that prioritized the reduction of confounding as a secondary analysis. Fourth, the study period included the first years of the COVID-19 pandemic, when health care was severely impacted by lockdowns. While there was a sharp decline in screening response rate in Flanders in 2020 (Figure S2 bottom), the effect on total screening coverage was limited (Figure S2 centre) and the analysis of pre-COVID years revealed that the association between language and the screening variables remained robust. Fifth, Flemish inhabitants may decline their screening invitation because they have been screened in a neighbouring country or region. This limitation is closely related to the modifiable areal unit problem (MAUP). Although our study did not fully eliminate the MAUP, a sensitivity analysis whereby border municipalities were excluded revealed that the associations between language and screening coverage and response rate were retained. Sixth, our study focused exclusively on FIT-based screening. In 2017, approximately 42.5 colonoscopies per 10,0000 inhabitants were carried out in Flanders for screening and therapeutic purposes combined, compared with 63 per 10,000 inhabitants in Wallonia and 40 per 10,000 in Brussels [54]. There may be geospatial differences in the likelihood that GPs recommend colonoscopy as a screening tool over FIT. This unmeasured variation may (in part) explain the observed disparities in screening response rate but not in total screening coverage, as the latter counts people who underwent colonoscopy for any purpose within the past 10 years as being up to date on their screening. Finally, Flanders has a specific screening protocol embedded in Belgium’s complex geopolitical and legal framework (e.g., the *Bestuurstaalwet*), limiting the external validity of our findings.

### Practical implications

Though legal language restrictions on governmental communication pose a supplementary challenge, language-based disparities in Flemish CRC screening may be overcome by some of the same interventions that target underscreened populations in general. (1) GPs are not bound by language laws and should be encouraged to discuss CRC screening with their patients in a language they understand [55]. (2) Mobile screening units can similarly circumvent language restrictions and offer easily accessible screening services to specific communities [56, 57]. (3) PSAs via social media can be translated by the platform or the internet browser, an advantage they have over leaflets and televised campaigns [58]. (4) Finally, sending reminders to non-responders may not mitigate language discordance but may impart a sense of import and urgency, prompting the recipient to seek more information [55].

## Conclusion

We demonstrate discrepancies in the colorectal cancer screening coverage and response rate in Flemish municipalities according to their proportion of secondary school pupils who do not speak Dutch at home. While individual-level research should further elucidate the effect of language on screening coverage and responses to screening invitation, policy interventions that aim to incentivize colorectal cancer screening should take into account that eligible individuals may experience linguistic challenges towards participation.

## Electronic supplementary material

Below is the link to the electronic supplementary material.

Supplementary Material 1

Supplementary Material 2

Supplementary Material 3

Supplementary Material 4

Supplementary Material 5

Supplementary Material 6

Supplementary Material 7

Supplementary Material 8

Supplementary Material 9

Supplementary Material 10

Supplementary Material 11

Supplementary Material 12

## Data Availability

The dataset supporting the conclusions of this article is included in the article’s supplementary material under the file name “Table S8– Source Data”. Data on CRC screening coverage and response rate are also available at: https://bevolkingsonderzoek.incijfers.be/ [21]. Data on language spoken at home and covariates are also available at: https://provincies.incijfers.be/databank [22].

## Abbreviations

AIC: Akaike information criterion
CI: Confidence interval
CRC: Colorectal cancer
CvKO: Centrum voor KankerOpsporing (Eng: Cancer Detection Centre)
DAG: Directed acyclic graph
fHb: Faecal haemoglobin
FIT: Faecal immunochemical test
IARC: International Agency for Research on Cancer
MAUP: Modifiable areal unit problem
VIF: Variance inflation factor
