# Supplementary material for: Language as a barrier to colorectal cancer screening in Flanders: an ecological study
Source: Arch Public Health. 2025 Mar 25;83:79. doi: 10.1186/s13690-025-01541-3 (PMC11934783; doi:10.1186/s13690-025-01541-3)
Supplement: Supplementary file 10 — Supplementary Material 10 [file 13690_2025_1541_MOESM10_ESM.docx]

# Supplementary Tables

**Table S1:** *Compliance to STROBE guidelines.*

This table is available as a separate .XLS file under the file name “Table S1 - Compliance to STROBE Guidelines”.

**Table S2a:** *Codebook of variables.*

| **Variable Name** | **Description** | **Remarks** | **Low-VIF Model** | **DAG Model** |
| --- | --- | --- | --- | --- |
| Total Screening Coverage | Total screening coverage (within programme + outside programme + valid exclusions) / all inhabitants of eligible age |  | Outcome | Outcome |
| Screening Response Rate | People screened within 1 year after invitation to official programme / all inhabitants of eligible age |  | Outcome | Outcome |
| Non-Dutch at Home | Secondary ed. students who don't speak Dutch at home (at the end of the school year) / all secondary ed. students | Collected in February of each year | Exposure | Exposure |
| Language at Home | % of children born in years X-(X-2) who are spoken to in language A by their mothers | Collected shortly after birth  See Table S1b for categories | Exposure | Exposure |
| Age Distribution | Percentual age distribution in 5-year categories based on population count of social security numbers | See Table S1b for categories | Yes | Yes |
| Sex Distribution | Proportion of males / all inhabitants |  | Response Rate only | Yes |
| Occupational Status | Proportion of inhabitants with the specified occupational status or source of income as defined in the Kruispuntbank Sociale Zekerheid (*Eng:* Central Database of Social Security) | See Table S1b for categories  Participation in the labour market is prioritized for people who fit >1 category (e.g. retired + working part-time) | Yes | No |
| Population Density | Population density calculated as the population with a social security number / area in km² |  | Total Coverage only | Yes |
| Educational Attainment | Proportional highest educational attainment level in inhabitants aged 25+ | See Table S1b for categories | No | Yes |
| Median Income | Median net taxable income of all taxpayers (excl. incomes of €0) in euro (€) | Per taxpayer; not per declaration | Yes | No |
| Child Deprivation Index | Children born into deprived families in year X and previous 2 years / all children born in those years |  | No | No |
| Chronical Disease(s) | Proportion of inhabitants with 1+ tatute(s) chronic disease |  | Response Rate only | No |
| Global Medical Record | Inhabitants with health insurance and a global medical record / all inhabitants with health insurance |  | Total Coverage only | No |
| Doctor Visits | Inhabitants with health insurance and 1+ doctor visit in past year / all inhabitants with health insurance |  | Response Rate only | No |
| Disabled | Proportion of inhabitants recognized by the "Directie-generaal Personen met een handicap" / 1000 adult inhabitants |  | No | No |
| Local Service Centres | Proportion of local service centres for the elderly / 10,000 inhabitants aged 65+ |  | No | No |
| Patients per GP Practice | Average number of patients per GP practice |  | Total Coverage only | No |
| Citizenship at Birth | % of inhabitants that has a particular nationality (aggregated into 3 zones) at birth | Non-Belgian citizenship is prioritized  See Table S1b for categories | No | Yes |
| Language Facilities | Binary variable indicating whether official communication in both Dutch and French is allowed in the municipality |  | Yes | Yes |
| Border Status (National) | Category denoting the adjacency of a municipality to the regions of Brussels and/or Wallonia | “Borders” is prioritized over “Near”,  Wallonia is prioritized over Brussels | Yes | No |
| Proximity to Walloon Border | Category denoting the adjacency of a municipality to the region of Wallonia |  | No | No |
| Proximity to Brusselian Border | Category denoting the adjacency of a municipality to the region of Brussels |  | No | No |
| Proximity to Dutch Border | Category denoting the adjacency of a municipality to the Netherlands |  | No | No |
| Proximity to French Border | Category denoting the adjacency of a municipality to France |  | No | No |
| Francophone Border Score | Integer (0-4) indicating the proximity to Wallonia, Brussels, and France | 2 points given for adjacency to a border, 1 point for being 1 municipality away from one | No | No |

*DAG = Directed Acyclic Graph; VIF = Variance Inflation Factor.*

**Table S2b:** *Codebook of variable subcategories.*

| **Category** | **Detail** | **Low-VIF Model** | **DAG Model** |
| --- | --- | --- | --- |
| *Age Distribution* |  |  |  |
| 50-54 Years |  | Total Coverage only | Response Rate only |
| 55-59 Years |  | Yes | Total Coverage only |
| 60-64 Years |  | Response Rate only | Yes |
| 65-69 Years |  | Yes | No |
| 70-74 Years |  | Total Coverage only | Response Rate only |
| *Occupational Status* |  |  |  |
| Wage-Earner Only |  | Response Rate only | No |
| Self-Employed Only |  | No | No |
| Jobseeker |  | No | No |
| Financial Aid Recipient |  | Response Rate only | No |
| (Early) Retired |  | Yes | No |
| *Educational Attainment* |  |  |  |
| Primary as Highest |  | No | Yes |
| Secondary as Highest |  | No | Yes |
| Tertiary as Highest |  | No | Yes |
| *Language at Home* |  |  |  |
| Dutch |  | Exposure | Exposure |
| French |  | Exposure | Exposure |
| Germanic | English / German | Exposure | Exposure |
| Eastern European | Russian / Polish / Romanian | Exposure | Exposure |
| Iberian Romance | Spanish / Portuguese | Exposure | Exposure |
| Middle-Eastern | Arabic / Turkish / Berber | Exposure | Exposure |
| Other Languages |  |  |  |
| *Citizenship at Birth* |  |  |  |
| Belgian |  | No | Total Coverage only |
| EU excl. Belgian |  | No | Response Rate only |
| Non-EU |  | No | Response Rate only |
| *Border Status (Main Analysis)* |  |  |  |
| Borders Wallonia | Borders at least one Wallonian municipality |  |  |
| Near Wallonia | Borders at least one Flemish municipality that borders at least one Wallonian municipality |  |  |
| Borders Brussels | Borders at least one Brusselian municipality |  |  |
| Near Brussels | Borders at least one Flemish municipality that borders at least one Brusselian municipality |  |  |
| Borders Brussels + Near Wallonia | Fulfills the criteria for both “Borders BR” and “Near WA” |  |  |
| Far | At least two municipalities away from both Brussels and Wallonia |  |  |
| *Border Status (Sensitivity Analysis)* |  |  |  |
| Borders | Borders the country or region in question |  |  |
| Near | Borders at least one Flemish municipality that borders the country or region in question |  |  |
| Far | At least two municipalities away from the country or region in question |  |  |

*DAG = Directed Acyclic Graph; VIF = Variance Inflation Factor.*

**Table S3:** *Characteristics of Flemish municipalities (n = 300), non-categorical variables.*

| **Description of Numerical Variable** | **Min.** | **Q1** | **Median** | **Mean** | **Q3** | **Max.** | **Missing** |
| --- | --- | --- | --- | --- | --- | --- | --- |
| Total Screening Coverage | 0.384 | 0.638 | 0.666 | 0.659 | 0.69 | 0.782 | 0 |
| Screening Response Rate | 0.136 | 0.499 | 0.539 | 0.529 | 0.575 | 0.703 | 0 |
| Proportion of Non-Dutch-at-Home Pupils | 0.01 | 0.043 | 0.07 | 0.108 | 0.126 | 0.737 | 21 |
| Proportion of 50-54-Year Olds | 0.047 | 0.072 | 0.076 | 0.076 | 0.079 | 0.092 | 0 |
| Proportion of 55-59-Year Olds | 0.054 | 0.07 | 0.075 | 0.075 | 0.079 | 0.112 | 0 |
| Proportion of 60-64-Year Olds | 0.044 | 0.062 | 0.066 | 0.066 | 0.07 | 0.133 | 0 |
| Proportion of 65-69-Year Olds | 0.038 | 0.053 | 0.057 | 0.058 | 0.061 | 0.12 | 0 |
| Proportion of 70-74-Year Olds | 0.029 | 0.044 | 0.048 | 0.049 | 0.052 | 0.115 | 3 |
| Proportion of Males | 0.467 | 0.491 | 0.496 | 0.496 | 0.501 | 0.603 | 0 |
| Proportion of Primary Education Highest | 0.146 | 0.297 | 0.328 | 0.322 | 0.358 | 0.444 | 1200 |
| Proportion of Secondary Education Highest | 0.185 | 0.291 | 0.309 | 0.305 | 0.321 | 0.391 | 1200 |
| Proportion of Tertiary Education Highest | 0.123 | 0.254 | 0.286 | 0.299 | 0.332 | 0.532 | 1200 |
| Median Net Taxable Income | 16,737 € | 20,138 € | 21,461 € | 21,531 € | 22,819 € | 28,320 € | 0 |
| Proportion of (Only) Wage-Earners | 0.161 | 0.325 | 0.345 | 0.336 | 0.357 | 0.4 | 0 |
| Proportion of (Only) Self-Employed | 0.038 | 0.07 | 0.081 | 0.085 | 0.095 | 0.21 | 0 |
| Proportion of Jobseekers | 0.005 | 0.011 | 0.014 | 0.014 | 0.017 | 0.039 | 0 |
| Proportion of Financial Aid Recipients | 0 | 0.001 | 0.002 | 0.003 | 0.003 | 0.018 | 0 |
| Proportion of People in (Early) Retirement | 0.141 | 0.191 | 0.203 | 0.204 | 0.214 | 0.392 | 0 |
| Average Number of Patients per Practice | 237 | 1300 | 1541 | 1639 | 1851 | 5631 | 15 |
| Population Density | 51 | 261 | 407.5 | 563.4 | 675.2 | 3369 | 0 |
| Child Deprivation Index | 0 | 0.051 | 0.078 | 0.092 | 0.118 | 0.386 | 303 |
| Proportion of People with Chronic Diseases | 0.072 | 0.105 | 0.118 | 0.119 | 0.131 | 0.196 | 1 |
| Proportion of People with Disabilities | 22.14 | 49.74 | 64.01 | 65.39 | 78.5 | 159.75 | 0 |
| Proportion of People with Global Medical Record | 0.426 | 0.799 | 0.85 | 0.833 | 0.887 | 0.954 | 0 |
| Proportion of People with 1+ Doctor Visit(s) | 0.66 | 0.84 | 0.86 | 0.853 | 0.88 | 0.93 | 0 |
| Local Service Centres per 10,000 Seniors | 0 | 0 | 0 | 1.416 | 2.7 | 9.2 | 0 |
| Proportion of Belgians at Birth | 0.405 | 0.857 | 0.918 | 0.886 | 0.944 | 0.988 | 0 |
| Proportion of Non-Belgian EU Citizens at Birth | 0.007 | 0.028 | 0.039 | 0.06 | 0.062 | 0.54 | 4 |
| Proportion of Non-EU Citizens at Birth | 0.004 | 0.025 | 0.039 | 0.054 | 0.063 | 0.276 | 5 |
| Proportion of French-at-Home Newborns | 0.032 | 0.746 | 0.842 | 0.786 | 0.895 | 1 | 1200 |
| Proportion of Dutch-at-Home Newborns | 0 | 0.006 | 0.015 | 0.063 | 0.039 | 0.742 | 1200 |
| Proportion of EN/GE-at-Home Newborns | 0 | 0.004 | 0.009 | 0.011 | 0.013 | 0.119 | 1200 |
| Proportion of PL/RO/RU-at-Home Newborns | 0 | 0.018 | 0.028 | 0.034 | 0.042 | 0.222 | 1200 |
| Proportion of SP/PO-at-Home Newborns | 0 | 0.004 | 0.008 | 0.011 | 0.014 | 0.08 | 1200 |
| Proportion of AR/TU/BE-at-Home Newborns | 0 | 0.013 | 0.026 | 0.043 | 0.053 | 0.304 | 1200 |

*Missing data (far right column) is expressed in counts. EN = English, GE = German, PL = Polish, RO = Romanian, RU = Russian, SP = Spanish, PO = Portuguese, AR = Arabic, TU = Turkish, BE = Berber.*

**Table S4:** *Most common languages used by mothers to speak to their children (0-2 years of age) in Flanders from 2013 to 2023. Data extracted from the website of Kind & Gezin (accessed 20/12/2024).*

|  | **2013** | **2014** | **2015** | **2016** | **2017** | **2018** | **2019** | **2020** | **2021** | **2022** | **2023** |
| --- | --- | --- | --- | --- | --- | --- | --- | --- | --- | --- | --- |
| **Dutch** | 74.5% | 74.3% | 73.5% | 72.8% | 71.3% | 71.0% | 70.0% | 70.0% | 70.6% | 69.3% | 68.6% |
| **French** | 5.2% | 5.3% | 5.4% | 5.6% | 5.7% | 5.8% | 5.9% | 6.4% | 6.5% | 6.6% | 6.5% |
| **Arabic** | 3.6% | 3.5% | 3.7% | 4.1% | 4.3% | 4.5% | 4.3% | 4.3% | 3.9% | 4.4% | 4.7% |
| **Turkish** | 3.0% | 3.1% | 2.9% | 2.7% | 2.8% | 2.7% | 2.7% | 2.6% | 2.3% | 2.0% | 2.0% |
| **Romanian** | 0.7% | 0.8% | 1.0% | 1.2% | 1.4% | 1.6% | 1.8% | 1.8% | 2.0% | 2.2% | 2.2% |
| **English** | 1.2% | 1.1% | 1.2% | 1.2% | 1.3% | 1.2% | 1.2% | 1.3% | 1.3% | 1.4% | 1.4% |
| **Polish** | 1.0% | 1.2% | 1.2% | 1.4% | 1.4% | 1.3% | 1.4% | 1.2% | 1.1% | 1.0% | 1.0% |
| **Berber** | 1.7% | 1.5% | 1.5% | 1.4% | 1.6% | 1.4% | 1.3% | 1.2% | 1.1% | 1.0% | 1.0% |
| **Other** | 9.0% | 9.3% | 9.3% | 9.8% | 10.2% | 10.5% | 11.3% | 11.1% | 11.1% | 12.0% | 12.0% |

**Table S5:** *Characteristics of Flemish municipalities (n = 300), categorical variables.*

| **National Border Status** | **Municipalities** | **N** | **Min.** | **Q1** | **Median** | **Mean** | **Q3** | **Max** |
| --- | --- | --- | --- | --- | --- | --- | --- | --- |
| *Screening Response Rate* |  |  |  |  |  |  |  |  |
| Far From Border | 204 | 1230 | 0.383 | 0.526 | 0.556 | 0.556 | 0.587 | 0.703 |
| Near Brussels | 11 | 66 | 0.256 | 0.453 | 0.484 | 0.473 | 0.519 | 0.575 |
| Borders Brussels | 8 | 48 | 0.198 | 0.297 | 0.333 | 0.332 | 0.386 | 0.448 |
| Borders Brussels + Near Wallonia | 4 | 24 | 0.136 | 0.198 | 0.284 | 0.282 | 0.369 | 0.438 |
| Near Wallonia | 38 | 222 | 0.437 | 0.501 | 0.529 | 0.530 | 0.556 | 0.656 |
| Borders Wallonia | 35 | 210 | 0.230 | 0.421 | 0.475 | 0.462 | 0.509 | 0.636 |
| *Total Screening Coverage* |  |  |  |  |  |  |  |  |
| Far From Border | 205 | 1230 | 0.559 | 0.656 | 0.675 | 0.676 | 0.698 | 0.782 |
| Near Brussels | 11 | 66 | 0.499 | 0.614 | 0.631 | 0.627 | 0.659 | 0.701 |
| Borders Brussels | 8 | 48 | 0.428 | 0.481 | 0.515 | 0.518 | 0.558 | 0.612 |
| Borders Brussels + Near Wallonia | 4 | 24 | 0.384 | 0.482 | 0.530 | 0.511 | 0.558 | 0.598 |
| Near Wallonia | 37 | 222 | 0.595 | 0.640 | 0.658 | 0.660 | 0.682 | 0.746 |
| Borders Wallonia | 35 | 210 | 0.507 | 0.574 | 0.618 | 0.614 | 0.653 | 0.721 |
| **Walloon Border** |  |  |  |  |  |  |  |  |
| *Screening Response Rate* |  |  |  |  |  |  |  |  |
| Borders | 35 | 210 | 0.230 | 0.421 | 0.475 | 0.462 | 0.509 | 0.636 |
| Near | 42 | 252 | 0.136 | 0.491 | 0.526 | 0.506 | 0.551 | 0.656 |
| Far | 223 | 1338 | 0.198 | 0.517 | 0.551 | 0.544 | 0.584 | 0.703 |
| *Total Screening Coverage* |  |  |  |  |  |  |  |  |
| Borders | 35 | 210 | 0.507 | 0.574 | 0.618 | 0.614 | 0.653 | 0.721 |
| Near | 42 | 252 | 0.384 | 0.631 | 0.654 | 0.646 | 0.680 | 0.746 |
| Far | 223 | 1338 | 0.428 | 0.649 | 0.673 | 0.668 | 0.695 | 0.782 |
| **Brusselian Border** |  |  |  |  |  |  |  |  |
| *Screening Response Rate* |  |  |  |  |  |  |  |  |
| Borders | 15 | 90 | 0.136 | 0.261 | 0.329 | 0.321 | 0.389 | 0.448 |
| Near | 17 | 102 | 0.256 | 0.450 | 0.481 | 0.470 | 0.515 | 0.591 |
| Far | 268 | 1608 | 0.279 | 0.514 | 0.546 | 0.544 | 0.579 | 0.703 |
| *Total Screening Coverage* |  |  |  |  |  |  |  |  |
| Borders | 15 | 90 | 0.384 | 0.490 | 0.539 | 0.525 | 0.562 | 0.619 |
| Near | 17 | 102 | 0.499 | 0.597 | 0.628 | 0.621 | 0.648 | 0.701 |
| Far | 268 | 1608 | 0.507 | 0.647 | 0.671 | 0.669 | 0.692 | 0.782 |
| **Dutch Border** |  |  |  |  |  |  |  |  |
| *Screening Response Rate* |  |  |  |  |  |  |  |  |
| Borders | 35 | 210 | 0.394 | 0.554 | 0.590 | 0.584 | 0.620 | 0.703 |
| Near | 40 | 240 | 0.437 | 0.538 | 0.572 | 0.570 | 0.599 | 0.691 |
| Far | 225 | 1350 | 0.136 | 0.488 | 0.530 | 0.513 | 0.561 | 0.666 |
| *Total Screening Coverage* |  |  |  |  |  |  |  |  |
| Borders | 35 | 210 | 0.531 | 0.663 | 0.681 | 0.682 | 0.706 | 0.773 |
| Near | 40 | 240 | 0.596 | 0.664 | 0.685 | 0.685 | 0.705 | 0.782 |
| Far | 225 | 1350 | 0.384 | 0.630 | 0.659 | 0.651 | 0.685 | 0.767 |
| **French Border** |  |  |  |  |  |  |  |  |
| *Screening Response Rate* |  |  |  |  |  |  |  |  |
| Borders | 10 | 60 | 0.308 | 0.442 | 0.484 | 0.474 | 0.526 | 0.573 |
| Near | 10 | 60 | 0.279 | 0.484 | 0.521 | 0.502 | 0.543 | 0.596 |
| Far | 280 | 1680 | 0.136 | 0.503 | 0.542 | 0.532 | 0.578 | 0.703 |
| *Total Screening Coverage* |  |  |  |  |  |  |  |  |
| Borders | 10 | 60 | 0.535 | 0.608 | 0.635 | 0.635 | 0.662 | 0.719 |
| Near | 10 | 60 | 0.507 | 0.657 | 0.675 | 0.661 | 0.686 | 0.746 |
| Far | 280 | 1680 | 0.384 | 0.639 | 0.666 | 0.660 | 0.691 | 0.782 |
| **Language Facilities** |  |  |  |  |  |  |  |  |
| *Screening Response Rate* |  |  |  |  |  |  |  |  |
| No | 288 | 1728 | 0.293 | 0.506 | 0.541 | 0.538 | 0.577 | 0.703 |
| Yes | 12 | 72 | 0.136 | 0.248 | 0.296 | 0.313 | 0.369 | 0.571 |
| *Total Screening Coverage* |  |  |  |  |  |  |  |  |
| No | 288 | 1728 | 0.467 | 0.643 | 0.668 | 0.664 | 0.691 | 0.782 |
| Yes | 12 | 72 | 0.384 | 0.506 | 0.530 | 0.524 | 0.554 | 0.706 |

**Table S6:** *Spearman’s rank correlation coefficients matrix of non-categorical variables considered for analysis. (n = 1800)*


*Dark green = significant after Bonferroni correction (p < 2.2E-16); green = significant after Bonferroni correction (2.2e-16 ≤ p < 1.15E-4); yellow = insignificant after Bonferroni correction (1.15E-4 ≤ p < 0.05); orange = insignificant before Bonferroni correction (p ≥ 0.05); red = complete autocorrelation.*

**Table S7:** *Beta coefficients and corresponding 95% CIs of DAG-compliant multiple linear regression models with the proportion of children addressed in a specific language by their mothers as the independent variable of interest. (n = 600; 300 municipalities over 2 years)*

| **Language Group** | **β (95% CI)** | |
| --- | --- | --- |
|  | *Response Rate* | *Total Coverage* |
| *Dutch* | 0.342 (0.305; 0.379) | 0.202 (0.181; 0.223) |
| *French* | -0.358 (-0.397; -0.319) | -0.213 (-0.238; -0.188) |
| *German, English* | -0.429 (-0.823; -0.035) | -0.381 (-0.628; -0.381) |
| *Russian, Polish, Romanian* | -0.252 (-0.402; -0.102) | -0.069 (-0.165; 0.027) |
| *Spanish, Portuguese* | -1.146 (-1.598; -0.694) | -0.368 (-0.656; -0.080) |
| *Arabic, Turkish, Berber* | 0.449 (0.327; 0.571) | 0.326 ± (0.250; 0.402) |
| *Other* | 0.311 (0.168; 0.454) | -0.148 (-0.241; -0.055) |

**Table S8:** *Z scores and p-values (between parentheses) of Dunn’s tests following significant Kruskal-Wallis tests to gauge differences in screening response rate and total screening coverage by proximity to each regional or national border.*

| **Z (*p*)** | **Response Rate** | **Total Coverage** |
| --- | --- | --- |
| *Walloon Border* |  |  |
| Borders - Near | -6.351 (*<1.00E-04*) | -6.437 (*<1.00E-04*) |
| Borders - Far | -15.124 (*<1.00E-04*) | -14.380 (*<1.00E-04*) |
| Near - Far | -7.707 (*<1.00E-04*) | -6.785 (*<1.00E-04*) |
| *Brusselian Border* |  |  |
| Borders - Near | -4.749 (*<1.00E-04*) | -4.920 (*<1.00E-04*) |
| Borders - Far | -16.209 (*<1.00E-04*) | -16.070 (*<1.00E-04*) |
| Near - Far | -10.469 (*<1.00E-04*) | -10.079 (*<1.00E-04*) |
| *Dutch Border* |  |  |
| Borders - Near | 2.645 (*0.0123*) | -0.375 (*1.000*) |
| Borders - Far | 14.437 (*<1.00E-04*) | 9.019 (*<1.00E-04*) |
| Near - Far | 11.720 (*<1.00E-04*) | 10.056 (*<1.00E-04*) |
| *French Border* |  |  |
| Borders - Near | 2.024 (*0.0645*) | -4.090 (*1.00E-04*) |
| Borders - Far | -6.778 (*<1.00E-04*) | -4.792 (*<1.00E-04*) |
| Near - Far | -3.966 (*1.00E-04*) | 0.891 (*0.559*) |

**Table S9:** *Processed source data.*

This table is available as a separate .XLS file under the file name “Table S9 – Source Data”.
