# Supplementary material for: Language as a barrier to colorectal cancer screening in Flanders: an ecological study
Source: Arch Public Health. 2025 Mar 25;83:79. doi: 10.1186/s13690-025-01541-3 (PMC11934783; doi:10.1186/s13690-025-01541-3)
Supplement: Supplementary file 11 — Supplementary Material 11 [file 13690_2025_1541_MOESM11_ESM.docx]

# Supplementary Figures


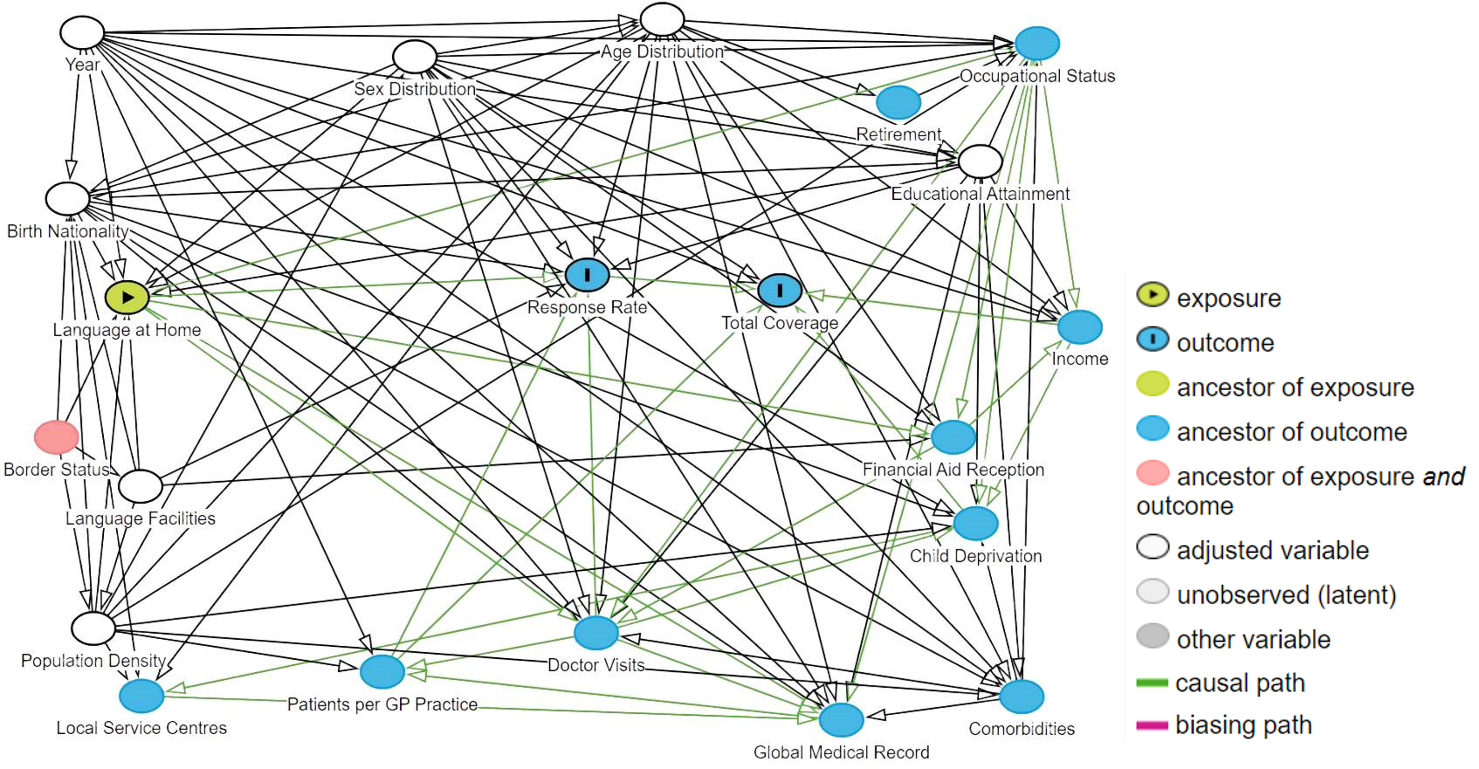


**Fig. S1:** *Directed acyclic graph (DAG) of all variables collected for the analysis of language at home and screening outcomes.*


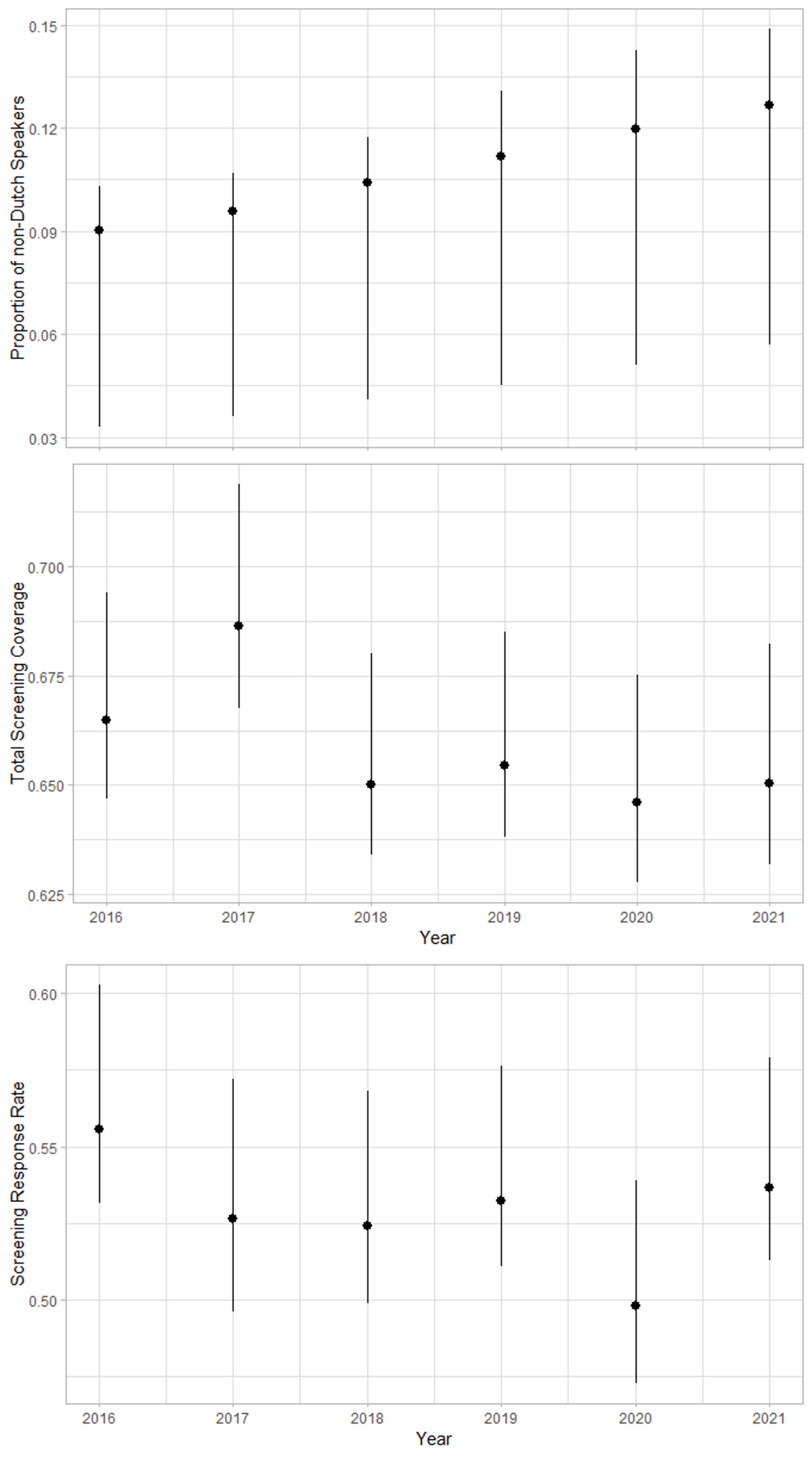


**Fig. S2:** *Means and interquartile ranges of the proportion of non-Dutch speakers (top), total screening coverage (centre), and screening response rate (bottom) across the study period.*


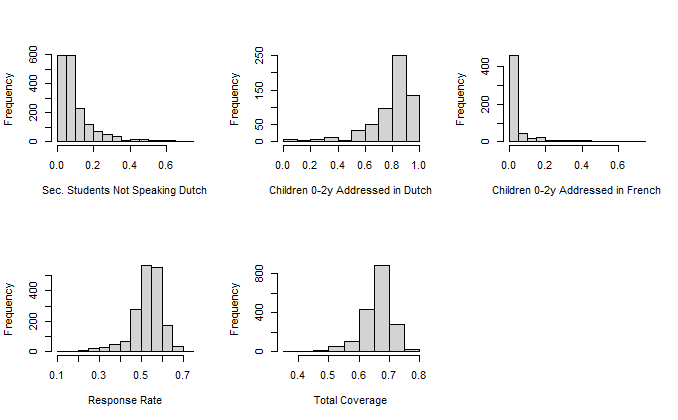


**Fig. S3:** *Frequency distribution histograms of the main exposure and outcome variables.*


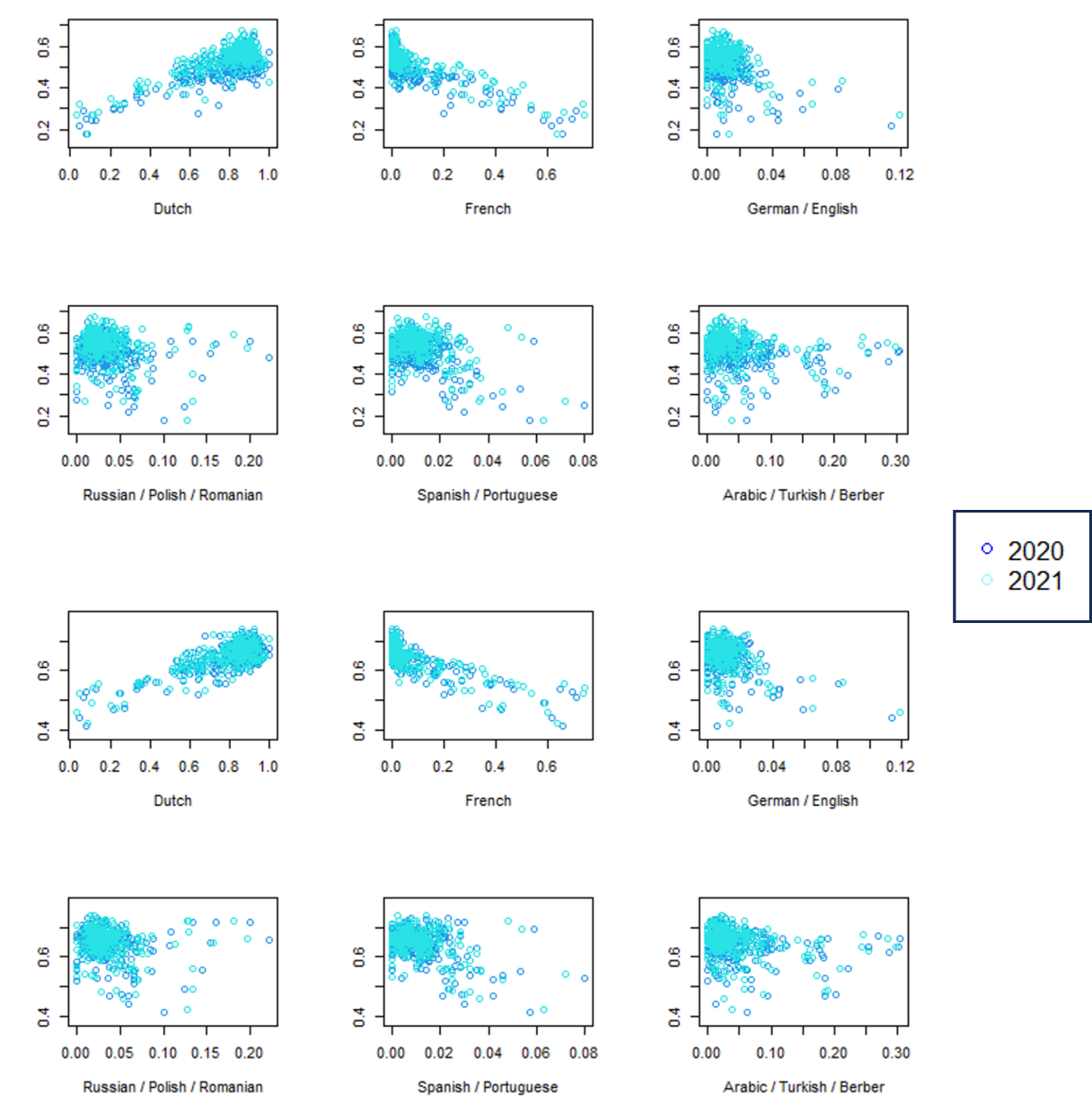


**Fig. S4:** *Scatterplots of screening response rate (top) and total screening coverage (bottom) by the proportion of children 0-2 years of age addressed in one of the specified languages by their mothers. (n = 600; 300 municipalities over 2 years)*


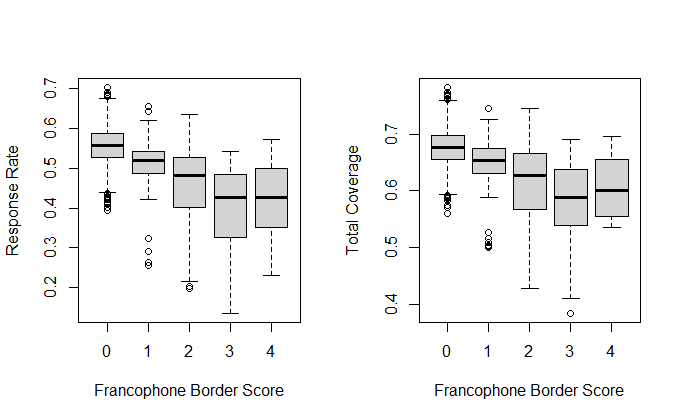


**Fig. S5:** *Boxplots of screening response rate (left) and total coverage (right) by Francophone border score.*


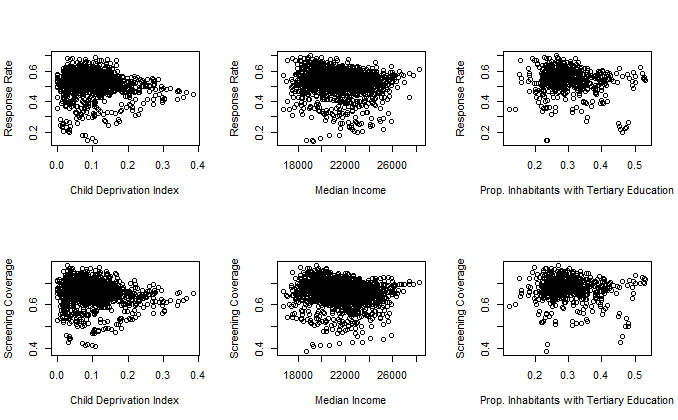


**Fig.S6:** *Scatterplots of screening response rate (top) and total screening coverage (bottom) by child deprivation index (left), median income (centre), and the proportion of inhabitants with a level of education higher than secondary school (right).*
